# Global and local effects in lipid-mediated interactions between peripheral and integral membrane proteins

**DOI:** 10.3389/fmolb.2025.1605772

**Published:** 2025-05-30

**Authors:** Tatiana K. Rostovtseva, David P. Hoogerheide, William A. Milhizer, Sergey M. Bezrukov

**Affiliations:** ^1^ Division of Basic and Translational Biophysics, Eunice Kennedy Shriver National Institute of Child Health and Human Development, National Institutes of Health, Bethesda, MD, United States; ^2^ Center for Neutron Research, National Institute of Standards and Technology, Gaithersburg, MD, United States

**Keywords:** tubulin, alpha-synuclein, gramicidin A, planar lipid membranes, ion channels, amphitropic proteins, protein-lipid interactions, lipid packing stress

## Abstract

Amphitropic proteins (APs) are a subfamily of water-soluble peripherally membrane-bound proteins that interact directly with the lipid membrane rather than with intrinsic membrane proteins and are therefore strongly influenced by membrane properties. When an AP interacts with a membrane containing an integral membrane protein, a ternary protein-lipid-protein system is created. Even in the absence of direct interactions between the amphitropic and integral proteins, the two proteins can affect each other by modifying lipid membrane properties, either at the global (i.e., whole-membrane) or local (i.e., confined to a small area around the bound or integrated protein) scale. These lipid-mediated protein-protein interactions are indirect and, therefore, difficult to elucidate; independent experimental data are required to report on each individual interaction to comprehend the whole system. Examples for which comprehensive data are available are remarkably rare. In this article, we describe how these difficulties could be surmounted by using the channel-forming integral membrane protein gramicidin A (grA) reconstituted in a planar lipid membrane and exposed to the amphitropic proteins dimeric tubulin or α-synuclein. Importantly, there are no known direct interactions between these APs and grA, thus revealing the role of the lipid membrane. Here, grA serves a dual role. First, grA reports on the global properties of the lipid membrane; grA results, combined with the well-understood tubulin-lipid interaction, yield a complete picture of the mutual effect of tubulin binding on the lipid membrane. Second, the presence of the grA conducting dimer alters the local membrane curvature and creates binding sites for tubulin in an otherwise inert membrane composition.

## 1 Introduction

The notion that lipids are not merely passive fillers of the space between membrane proteins, but actively interacting with them, controlling protein conformational transitions and affecting their function, is now well-accepted (Maer et al., 2022; Levental and Lyman, 2023). Proteins define lipids’ structural and compositional distributions in cell membranes, and, conversely, lipids influence localization and properties of proteins. While some protein-lipid interactions involve chemical interactions between lipids and specific protein residues, others involve indirect interaction through perturbation of the lipid bilayer by proteins, thereby affecting membrane properties such as curvature. This has been shown for different families of integral membrane proteins (IMPs) (Sprong et al., 2001; Phillips et al., 2009; Corradi et al., 2019; Thakur et al., 2023), especially ion channels and transporters (Sunshine and McNamee, 1994; Nielsen et al., 1998; Kloda et al., 2007; Cordero-Morales and Vasquez, 2018). In most cases, membrane lipid composition influences ion channel gating properties and ligand binding (Gruner, 1985; Keller et al., 1993; Bezrukov, 2000; van den Brink-van der Laan et al., 2004; Rostovtseva et al., 2006; Sachs, 2010; Elinder and Liin, 2017; Mlayeh et al., 2017; Maer et al., 2022; Beverley and Levitan, 2024), by affecting channels’ protein-lipid interface.

The functions of conventional ion channels and transporters in the plasma membrane are regulated by ligands, or small molecules directly interacting with the receptor binding site of channel protein [see e.g., Table 1 in a recent review (Cordero-Morales and Vasquez, 2018)]. Less studied are amphiphilic small molecules that modulate ion channel behavior, not by directly interacting with channel-forming proteins, but indirectly by modifying the lipid membrane properties (Cordero-Morales and Vasquez, 2018). Not surprisingly, the effect of changes in the bilayer pressure on membrane proteins, or the so-called “force-from-lipid” effect, has been mostly studied on mechanosensitive or stretch-activated ion channels (Sukharev et al., 2001; Gullingsrud and Schulten, 2004; Sachs, 2010; Teng et al., 2015; Gerhold and Schwartz, 2016). A purified bacterial mechanosensitive channel of large conductance (MscL), for example, remained mechanosensitive even after reconstitution into model planar membranes (Martinac et al., 1987). However, determination of the precise molecular mechanism of how lipids modulate ion channel gating—through direct or indirect interaction, activating or deactivating—is a challenging task due to the structural complexity of the plasma membrane ion channels, such as mechanotransduction channels, glutamate receptor channels, transient receptor potential (TRP) channels, or Ca^2+^-activated large-conductance K^+^ (BK) channels. To overcome these apparent difficulties, the small ideally cation-selective channel gramicidin A (grA) has been extensively studied since the early 1980s (Elliott et al., 1983; Lundbaek and Andersen, 1999; Suchyna et al., 2004; Andersen et al., 2005; Lundbaek et al., 2010; Weinrich et al., 2017). As will be discussed later, the conductance and characteristic lifetime of this small dimeric channel respond exceptionally well to the changes in the bilayer charge, thickness, and lateral pressure distribution and are thus valuable for reporting on the channel’s lipid environment.

**TABLE 1:** Tubulin effects on grA channel lifetime and conductance in PC and PE membranes.

Lipid	Bilayer thickness^a^, nm	Area per lipid, nm^2^	Lifetime, s		Conductance, pS	
			No tubulin	Tubulin (30 nM)	No tubulin	With tubulin (30 nM)
DOPC (C-18:1)	3.67(^b^)	0.724	4.5 ± 1.0	3.2 ± 0.9	21.8 ± 0.4	21.1 ± 0.6
DOPE/DOPC (3:1) (C-18:1)	3.83(^c^)	0.645	0.4 ± 0.05	2.3 ± 0.1	34.2 ± 0.5	28.1 ± 0.8
diC (22:1) PC	4.43(^b^)	0.693	0.11 ± 0.01	0.125 ± 0.08	19.1 ± 3.8	16.4 ± 4.3
DPhPC C-18-(CH3)_4_	3.64(^d^)	0.805	7.9 ± 0.4	39.5 ± 3.3	22 ± 0.7	18.9 ± 1.0

GrA channel parameters were measured in 1 M KCl at pH 7.4. Uncertainties are 68% confidence intervals of the mean derived from multiple repeated measurements [Adapted with permission from Rostovtseva et al. (2024)].

^a^ The phosphate-phosphate thickness.

^b^ From Kucerka et al. (2005).

^c^ From Rand et al. (1990).

^d^ From Tristram-Nagle et al. (2010).

Amphitropic proteins (AP) are a subfamily of peripherally membrane-bound proteins that interact directly with the lipid membrane rather than with intrinsic membrane proteins and are therefore strongly influenced by lipid composition (Anderluh and Lakey, 2008; Prinz, 2010; Holthuis and Menon, 2014). APs are involved in various cell signaling pathways and lipid trafficking (Cho and Stahelin, 2005; Monkhouse and Deane, 2024). However, due to the transient nature of AP-membrane binding, the molecular mechanisms of these interactions are generally poorly understood. The effects of structurally and functionally diverse amphiphiles on the properties of bilayer membranes were described by Olaf Andersen and colleagues as changes in lipid intrinsic curvature, which reflects the attractive and repulsive forces between lipid molecules (Lundbaek and Andersen, 1999; Andersen and Koeppe, 2007; Lundbaek et al., 2010). These forces include electrostatic interactions between lipid headgroups and hydrophobic interactions between the acyl chains and depend on the “shape” of the lipid molecule and the lipid headgroup dipole charge. For instance, the bilayers formed from lamellar dioleoyl-phosphatidylcholine (DOPC) and nonlamellar dioleoyl-phosphatidylethanolamine (DOPE) present a striking difference in the lipid packing stress profile: the transition from DOPC to DOPE was estimated as the change of *ΔP* ∼ 100 atm or 10^7^ pascals in the lateral pressure in the hydrocarbon area of a planar membrane (Bezrukov, 2000; Gullingsrud and Schulten, 2004). This could be a result of the decrease of the cross-sectional area per lipid molecule from 0.72 nm^2^ in lamellar DOPC (Kucerka et al., 2005) to 0.645 nm^2^ in nonlamellar DOPE/DOPC (3:1 mol/mol) mixture (Rand et al., 1990). This, in turn, causes some difference of ≈0.16 nm in the hydrophobic thickness between DOPC and DOPE bilayers (Rand et al., 1990; Kucerka et al., 2005) (Table 1).

**FIGURE 1:**
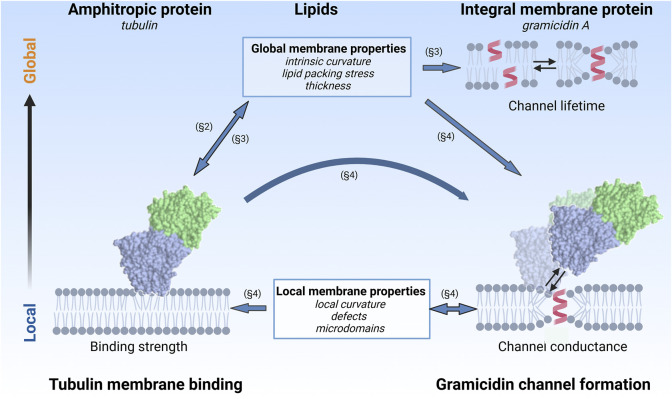
Lipid-mediated protein-protein interactions between an amphitropic protein, dimeric tubulin, and an integral membrane protein, gramicidin A. Both global and local membrane properties play a role and are reported on by the lifetime and conductance of the grA channel, respectively. Block arrows denote a direction of influence and do not indicate chemical equilibrium. Sections in which the various interactions are discussed are denoted by §. The lipid influence on gramicidin indicated in the upper right corner of the cartoon was shown to be reciprocal (Szule and Rand, 2003; Hassan-Zadeh et al., 2017). However, the oppositely directed effect happens only at much higher gramicidin concentrations of one or larger molar percent and is thus not relevant to our study. Created in BioRender.com.

When APs interact with a membrane containing IMPs, a ternary protein-lipid-protein system is created (Figure 1). Even in the absence of direct interactions between APs and IMPs, the two proteins can affect each other by modifying lipid membrane properties, either at the global (i.e., whole-membrane) or local (i.e., confined to a small area around the bound or integrated protein) scale. These lipid-mediated protein-protein interactions are indirect and, therefore, difficult to study; multiple experimental approaches are required to clarify individual interactions contributing to the whole system’s behavior.

In this review, we describe how these difficulties could be surmounted by using a system of an IMP, the grA channel, reconstituted in a planar lipid membrane (PLM) in the presence of an AP, the water-soluble dimeric tubulin. Importantly, though there are no known direct interactions between tubulin and grA, tubulin absorption to the membrane surface modifies the channel behavior, thus revealing the role of the lipid membrane. We leverage our detailed understanding of the tubulin-lipid interaction mechanism, as well as the self-reporting properties of the grA channel, to understand how tubulin affects the properties of the lipid membrane. We also discuss here the converse relationship, where the presence of grA conducting dimer alters the local curvature and creates binding sites for tubulin in an otherwise inert membrane composition. We also compare the effect of the globular protein tubulin on the grA channel with that of another well-studied AP, the intrinsically disordered α-synuclein (αSyn).

The general structure of this review is shown in Figure 1. Global and local effects are differentiated by their vertical position in the figure, while the three components (AP, lipids, IMP) are distributed horizontally. Arrows indicate the direction of effects (not chemical equilibrium). In Section 2, we discuss the role of lipid composition on tubulin membrane binding. Section 3 delves into the effect of tubulin on the lipid membrane properties, as reported by the grA lifetime. Section 4 explores what can be learned about global and local membrane properties from the grA conductance. The use of flickering in the grA conductance to detect the presence of tubulin is discussed, with implications for the creation of local binding sites for APs around IMPs, in case the latter significantly distorts the lipid membrane structure. Finally, in Section 5, we compare the effects of αSyn and tubulin on grA channel properties.

## 2 Tubulin binding depends on lipid composition

### 2.1 Tubulin

Tubulin is a prime example of an abundant cytosolic AP, which has multiple functions in a cell. The major role of dimeric tubulin is to serve as a building block of microtubules (Nogales et al., 1998). It is also found to be associated with cellular, particularly mitochondrial, membranes (Bernier-Valentin et al., 1983; Carre et al., 2002; Rostovtseva et al., 2018) where dimeric tubulin interacts with and regulates the Voltage Dependent Anion Channel (VDAC), the most abundant metabolite channel at the mitochondrial outer membrane (MOM). Tubulin is a compactly folded globular protein of 110-kDa molecular weight with a well-defined α/β-heterodimeric structure (Nogales et al., 1998). Each of its α- and β-subunits has a disordered, negatively charged C-terminal tail (CTT) exposed at the protein surface. Using an *in vitro* system of VDAC reconstituted to the PLM, it was shown that tubulin could efficiently regulate VDAC conductance by the highly negatively charged disordered CTT of either the α- or β-subunit (Rostovtseva et al., 2018; Rostovtseva et al., 2021). Under an applied transmembrane potential, the CTT is stochastically captured by the VDAC pore and eventually escapes, resulting in a transient decrease of channel conductance by 60% and a reversal of the ionic selectivity from anionic to cationic (Gurnev et al., 2011). This led to the proposal of a multistep model of tubulin-VDAC interaction where the first step is tubulin binding to the membrane, followed by a partial and reversible block of VDAC pore by anionic tubulin CTT driven by the applied potential (Rostovtseva et al., 2021). Experiments with isolated mitochondria (Monge et al., 2008; Rostovtseva et al., 2008) and human hepatoma live cells (Maldonado et al., 2010; Maldonado et al., 2013) further confirmed that tubulin could modulate mitochondrial metabolism by interacting with VDAC *in vivo*.

Importantly, *in vitro* studies showed that the tubulin-VDAC interaction depends strongly on membrane lipid composition (Rostovtseva et al., 2012). The mechanism of tubulin-VDAC interaction is an important example of how membrane lipids modulate ion channel conductance indirectly by changing the effective concentration of membrane-bound channel-regulating AP.

### 2.2 Tubulin membrane interactions

**FIGURE 2:**
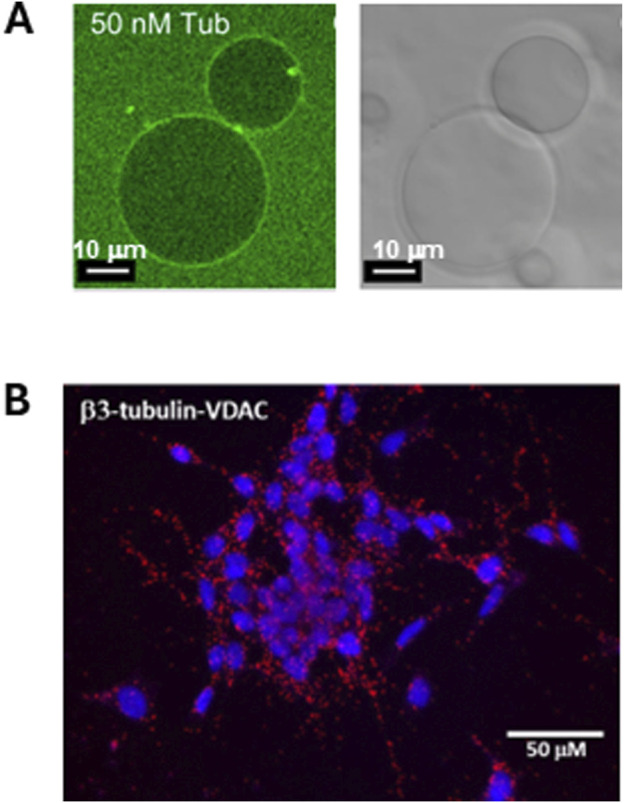
Dimeric tubulin is associated with liposome membranes and with the mitochondrial outer membranes. **(A)** Tubulin-488 binds to the surface of DOPE-containing giant unilamellar liposomes (GUVs). Left panel: confocal image of GUVs prepared from DOPC/DOPE in a 7:3 M ratio in the presence of 50 nM (M = mol/L) of bovine brain tubulin labeled with HiLyte Fluor 488. The GUVs in the right panel are shown in white light. Adapted with permission from Rostovtseva et al. (2012). © 2012 ASBMB. Currently published by Elsevier Inc; originally published by American Society for Biochemistry and Molecular Biology. **(B)** β3-Tubulin is colocalized with VDAC1 in neuroblastoma cells. The image of Duolink *in situ* proximity ligation assay (PLA). Interaction between β3-tubulin and VDAC is indicated by a red positive reaction. Adapted with permission from Rostovtseva et al. (2018).

The first observations of the reversible association of tubulin with lipid membranes, and estimates of the binding constants, were made in 1980s (Caron and Berlin, 1979; Bernier-Valentin et al., 1983), thus establishing tubulin’s identity as an amphitropic protein. Preferential binding to nonlamellar phosphatidylethanolamine (PE) lipids was first observed as an increase in microtubule assembly at surfaces containing PE lipids (Hargreaves and McLean, 1988). Later, increased tubulin binding to PE-containing membranes was demonstrated using the confocal microscopy of giant unilamellar vesicles with fluorescently labeled tubulin (Rostovtseva et al., 2012) (Figure 2A). It is particularly noteworthy that phosphatidylcholine (PC) and PE lipids are the main components of the mitochondrial outer membrane (Horvath and Daum, 2013). Besides the detection of mitochondrial-associated tubulin in the neuroblastoma cells (Carre et al., 2002) (Figure 2B), the physiological role(s) of tubulin binding to the mitochondrial membranes remained questionable until the new role of free dimeric tubulin as a potent regulator of mitochondrial respiration through its interaction with VDAC at the MOM was discovered (Monge et al., 2008; Rostovtseva et al., 2008; Maldonado et al., 2010).

**FIGURE 3:**
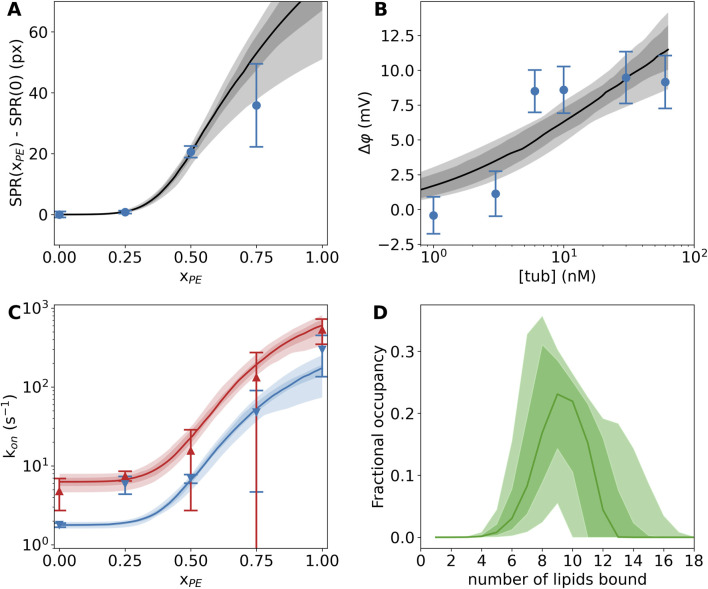
Tubulin binding to lipid membranes depends strongly on the PE lipid concentration. **(A)** Surface plasmon resonance shows significantly more binding at 600 nM tubulin concentration in DOPE-rich membranes than pure DOPC membranes. **(B)** Bilayer overtone analysis reveals the slow increase in transmembrane potential when tubulin is bound to one side of the membrane at increasing concentrations. **(C)** A single VDAC channel embedded in a lipid membrane is exquisitely sensitive to the presence of tubulin. Red and blue curves show the rates of blockage at 25 mV and 20 mV applied transmembrane potential, respectively. **(D)** The multisite interaction model encodes the lipid interaction site occupancy. The distribution of occupied sites is shown here; on average, about nine lipids are bound to each tubulin molecule. In all panels, the dark (light) shaded areas show 68% (95%) confidence intervals, while the solid lines show the median model prediction. Error bars on data are the standard error of the mean from multiple independent experiments. Adapted with permission from Hoogerheide et al. (2017). Copyright (2017) National Academy of Sciences.

A recent study of tubulin binding to PE-rich membranes utilized three biophysical techniques—surface plasmon resonance (SPR), bilayer overtone analysis (BOA), and electrophysiology using the blockage rate of a single VDAC channel as a probe for the tubulin concentration—to reveal that even for a large globular protein like tubulin, the observed membrane binding constant depends strongly on the experimental design (Hoogerheide et al., 2017). For example, SPR (Figure 3A) is performed on a solid supported membrane with a large area relative to the buffer volume, while BOA (Figure 3B) and electrophysiology experiments (Figure 3C) are performed with reconstituted lipid membranes with a small active area relative to the bathing buffer volume. Thus, it is unsurprising that experimental results appear to differ across these techniques, because the membrane binding site(s) may have multiple lipid contacts and interact with a substantial area of the target membrane. These disparate data can be collectively understood using a binding model that assumes several equivalent lipid binding sites (Mosior and McLaughin, 1992) and accounts for the differing geometries of the measurement cells for each technique. Applying such an analysis to the SPR (Figure 3A), BOA (Figure 3B), and electrophysiology (Figure 3C) data, the average number of lipid-protein binding contacts was found to be ≈9, albeit broadly distributed (Figure 3D), while the average strength of binding per binding site, or the molar partition coefficient, was found to be K≈0.72 M (M = mol/L) for PE lipids.

**FIGURE 4:**
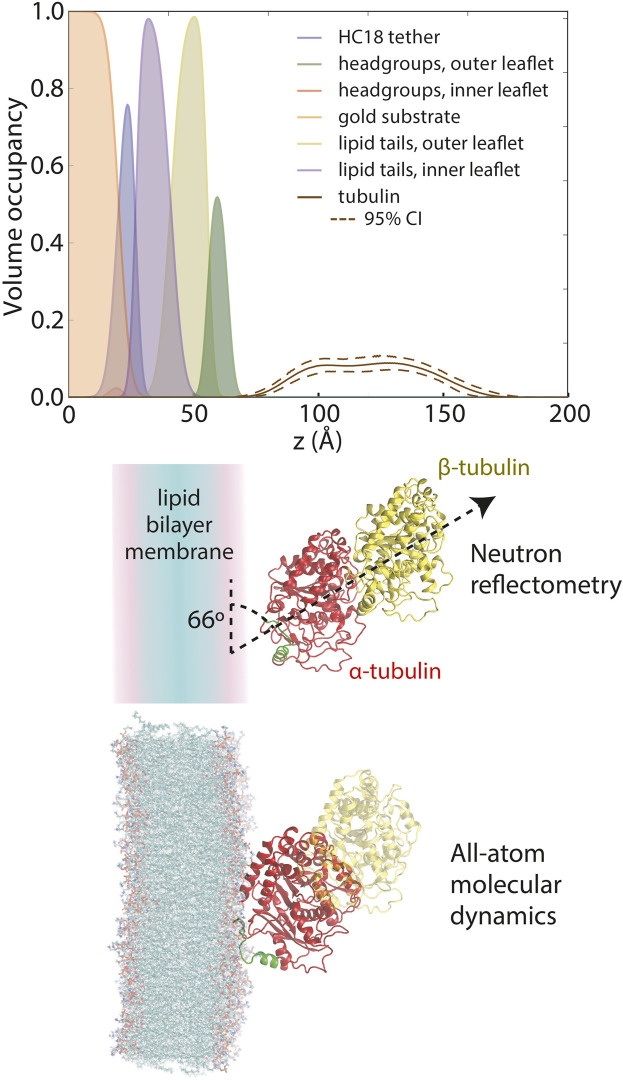
Composition space model for tubulin bound to a 1:1 DOPC:DOPE lipid membrane at 600 nM solution concentration, as derived from neutron reflectivity experiments. Adapted with permission from Hoogerheide et al. (2017). Copyright (2017) National Academy of Sciences.

The structural basis for the observed lipid dependence was determined using a combination of neutron reflectometry (NR) and molecular dynamics (MD) simulations (Hoogerheide et al., 2017). The sensitivity of neutron scattering techniques to light elements allows the decomposition of a measured reflectivity pattern into lipid and protein components. The resulting distributions of headgroups, acyl chains, and bound tubulin are shown in Figure 4 (top panel). In this experiment, tubulin is present at a density of about 1 tubulin molecule per 300 surface lipids. Notably, tubulin is fully peripheral, sitting at the membrane surface without penetrating the acyl chain region of the bilayer. At this low tubulin surface density, bilayer thinning is miniscule; in the context of the NR model, the change in bilayer thickness is $-0.08_{-0.06}^{+0.05}\;Å$ (68% CI); the change is not significant at the 95% confidence level.

**FIGURE 5:**
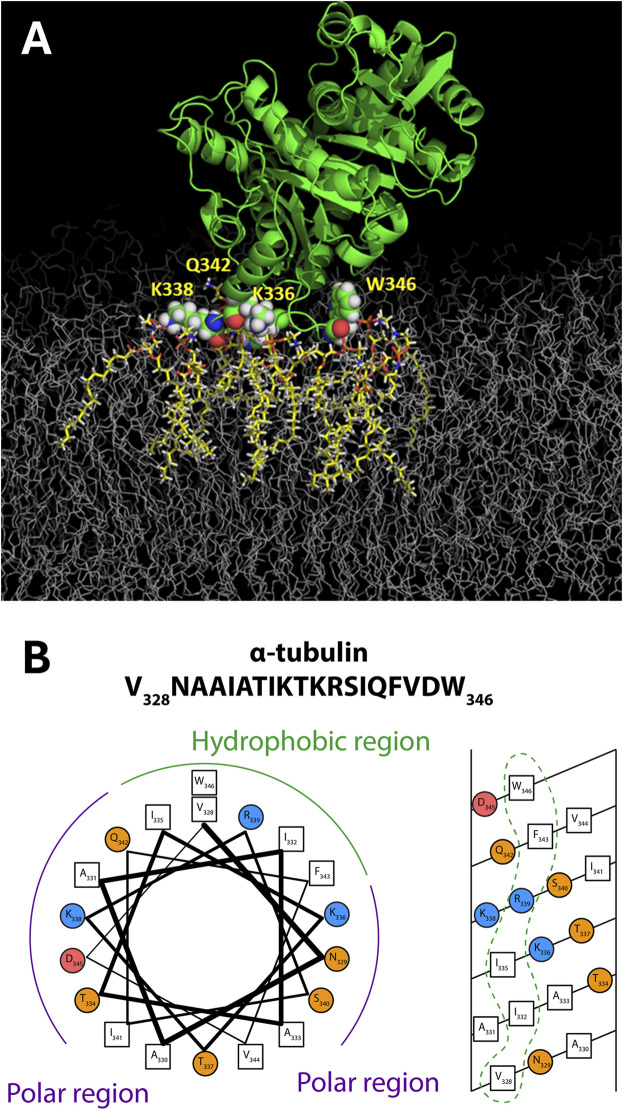
Tubulin membrane-binding domain. **(A)** Orientation of α-tubulin on a DOPE membrane surface from all-atom MD simulations on the ANTON2 platform. **(B)** The binding motif appears to have amphipathic helical properties. Adapted with permission from Hoogerheide et al. (2017). Copyright (2017) National Academy of Sciences.

When constrained by the known crystallographic structure of the tubulin heterodimer, further analysis revealed that the NR data are most consistent with a tilt angle of about 66° between the tubulin dimeric axis and the membrane surface (Figure 4, middle panel); however, NR could not determine which tubulin subunit was bound. All-atom MD simulations (Hoogerheide et al., 2017) established that only the α-subunit of the tubulin heterodimer can bind to a pure PE membrane (Figure 5A), via a highly conserved amphipathic helix (Figure 5B). The structure of the tubulin-lipid complex is remarkably similar to that obtained by NR (Figure 4, bottom panel). Notably, pure PC membranes were unable to stably bind either tubulin subunit in the simulations, consistent with the binding assays (Figure 3).

Together, the neutron reflectometry, molecular dynamics, and binding assays establish tubulin as a peripheral membrane protein via a α-helical binding domain. Thus, tubulin is an AP with a preference for PE-rich membrane compositions.

## 3 Tubulin redistributes the lateral pressure of lipid packing

### 3.1 GrA as a reporter on lipid membrane properties

The grA channel is a small ion channel formed by the trans-bilayer association of two grA monomers from each lipid monolayer into a conducting dimer (for a comprehensive review see, e.g., Andersen et al., 2005). It is a single-stranded β^6,3^-helical dimer whose structure is known at atomic resolution (Arseniev et al., 1985; Allen et al., 2003). The length of the conducting grA dimer, at ≈2.2 nm, is much less than the hydrophobic thickness of common lipid bilayers (Elliott et al., 1983). Therefore, channel formation requires locally bending the two lipid monolayers towards each other, creating a disjoining force on the channel that varies with lipid membrane properties such as chemical composition, hydrophobic thickness, membrane curvature, lipid packing stress, etc. Analysis of the observable channel parameters—its ionic conductance and the average lifetime of the conducting, dimeric state—thus reveals changes in the lipid bilayer environment. Importantly, grA as an IMP is thus “self-reporting” on its lipid environment.

**FIGURE 6:**
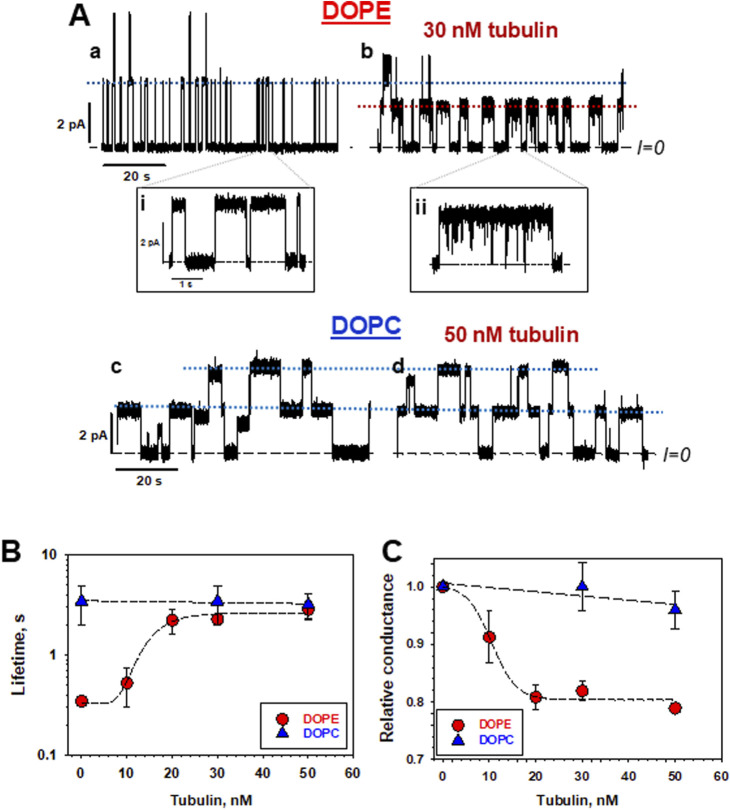
Tubulin affects grA channel parameters in DOPE, but not in DOPC membranes. **(A)** Current traces of grA channels in DOPE and DOPC membrane before (traces a and c) and after (traces b and d) addition of 30 nM and 50 nM tubulin, respectively. Tubulin notably increases grA lifetime and decreases channel conductance in DOPE membranes. Tubulin also induces fast current flickering that can be better seen at a finer time scale in inset (ii) in comparison with the control trace in inset (i). 50 nM of tubulin does not change grA channel parameters in DOPC membranes appreciably. The applied voltage was 100 mV. Tubulin was added to the cis compartment. Current records were digitally filtered using an averaging time of 10 ms. Dashed lines indicate zero current level, and dotted lines indicate the currents through single (or double, as in panel c) grA channels. The medium consisted of 1 M KCl buffered with 5 mM HEPES at pH 7.4. **(B,C)** In DOPE membranes, tubulin increases grA lifetime **(B)** and decreases conductance **(C)** in a dose-dependent manner that displays saturation at about 20 nM tubulin concentration. Tubulin has virtually no effect on channel lifetime and conductance in DOPC membranes. Channel conductance is given as its ratio in the presence of tubulin to that in the absence of tubulin. Uncertainties are 68% confidence intervals derived from multiple repeated measurements. Adapted with permission from Rostovtseva et al. (2024). © 2024 by the authors. Licensee MDPI, Basel, Switzerland.

Olaf Andersen and colleagues demonstrated in a number of their works that grA lifetime could be a reliable measure of the changes in bilayer intrinsic curvature, the repulsion/attraction forces between lipid headgroups, hydrophobic thickness, and several other parameters [for comprehensive reviews see (Lundbaek et al., 2010; Maer et al., 2022)]. The ≈10 times difference in grA lifetime between pure DOPC and DOPE membranes is convincing evidence of the high sensitivity of grA dissociation kinetics to the bilayer mechanics (Rostovtseva et al., 2008; Rostovtseva et al., 2024) (Figure 6A). GrA lifetime exponentially decreases with the increase of DOPE/DOPC ratio in lipid mixture (Rostovtseva et al., 2008).

A clear demonstration of the effect of hydrophobic thickness on grA lifetime is shown in Table 1, where the grA lifetime decreases with even a small increase of the bilayer thickness in monounsaturated PC bilayers (Huang, 1986; Lundbaek, 2006). The small difference of ≈0.16 nm in the thickness between DOPC (Kucerka et al., 2005) and bilayers of DOPE/DOPC (3:1) mixtures (Rand et al., 1990) (a pure nonlamellar DOPE does not form liposomes) (Table 1), can only partially contribute to the 10-fold difference in grA lifetime in these bilayers. It was also shown that channel lifetime decreased ≈40 times, from 4.5 s to 0.11 s, if bilayer thickness increased by 0.76 nm when PC with C(18:1) acyl chain in DOPC was replaced by PC with longer acyl chain C(22:1) in dier-ucoylphosphatidylcholine (diC(22:1)PC), respectively (Table 1) (Rostovtseva et al., 2024).

Hydrophobic thickness, however, is not the only determinant of the grA lifetime. The grA lifetime was also changed when monounsaturated acyl chains in DOPC lipid were replaced with phytanoyl chains in diphytanoyl-PC (DPhPC) (Rostovtseva et al., 2024): the lifetime in DPhPC was ≈2 times longer (≈8 s) than in DOPC (4.5 s), despite the thickness of both bilayers’ being essentially the same at 3.64 nm and 3.67 nm, respectively (Kucerka et al., 2005; Tristram-Nagle et al., 2010) (Table 1). These results can be understood by the observations of Tristam-Nagle et al. (2010) obtained using x-ray and neutron scattering and water permeability measurements on unilamellar vesicles. The authors suggested that from the biophysical perspective, DPhPC belongs to a different family of lipids than phosphatidylcholines with linear chain hydrocarbon chains. Notably, the bending modulus of DPhPC was 30% smaller than that of DOPC (Tristram-Nagle et al., 2010). Thus, the grA lifetime reports on the mechanical properties of the membrane, which include a contribution from the associated bilayer deformation energy or the “phenomenological bilayer spring constant” (Lundbaek and Andersen, 1999; Lundbaek et al., 2010), membrane surface bending, the hydrophobic thickness, and the lipid packing stress. We prefer to use a more broad term of lipid packing stress (Bezrukov, 2000; van den Brink-van der Laan et al., 2004) which includes the energetics of hydrophobic inclusions, such as those introduced by protein-lipid interactions, and the energetics of spontaneous formation of non-lamellar local structures. In this framework, the increase of grA lifetime corresponds to a reduction in the bilayer deformation energy for the formation of the grA dimer in response to the change of the lipid packing stress (Rostovtseva et al., 2008; Weinrich et al., 2009).

### 3.2 Effect of tubulin on membrane properties reported by grA

As discussed previously, dimeric tubulin preferentially binds to DOPE membranes. grA is the exemplary molecular probe to test if bound tubulin changes lipid bilayer properties (Figure 1). Indeed, the addition of tubulin to the DOPE membranes resulted in a change of grA channel parameters. Channel lifetime increased ≈10 times with the addition of 30 nM tubulin (Figures 6A,B), and conductance decreased by ≈20% (Figure 6C) in DOPE membranes. Conversely, in DOPC membranes, both parameters remained virtually unchanged (Rostovtseva et al., 2024). Similarly to DOPC membranes, tubulin did not change the grA lifetime in diC(22:1) membranes (Table 1), even though the grA lifetime in the membranes of the 18:1 PC (DOPC) lipids is 10 times smaller than that in 22:1 PC lipids. Channel conductance changed less dramatically than the lifetime, but, in DOPE membranes, the presence of tubulin produced rapid current fluctuations, with characteristic time ∼100 μs (compare traces in insets *i* and *ii* in Figure 6A). These results, obtained by using grA as a molecular probe, independently confirm tubulin’s preference for binding to DOPE lipids, as described in Section 2.

Most striking was the effect of tubulin on grA lifetime in DPhPC membranes: in the presence of 30 nM tubulin, the lifetime increased ≈5 times, similar to its effect in DOPE membranes (Table 1), despite presenting a PC headgroup at the membrane surface. Based on these data, we can suggest that tubulin-membrane binding depends not on specific interactions of tubulin with lipid headgroups, but rather on its lipid-dependent ability to distort the headgroup packing at the membrane surface and thus redistribute the lateral pressure of lipid packing as depicted by pathways §2 and 3 in Figure 1. Importantly, this occurs without a significant change in the hydrophobic thickness, as observed by NR (Section 2.2).

## 4 Global and local membrane properties derived from the grA channel lifetime and conductance

**FIGURE 7:**
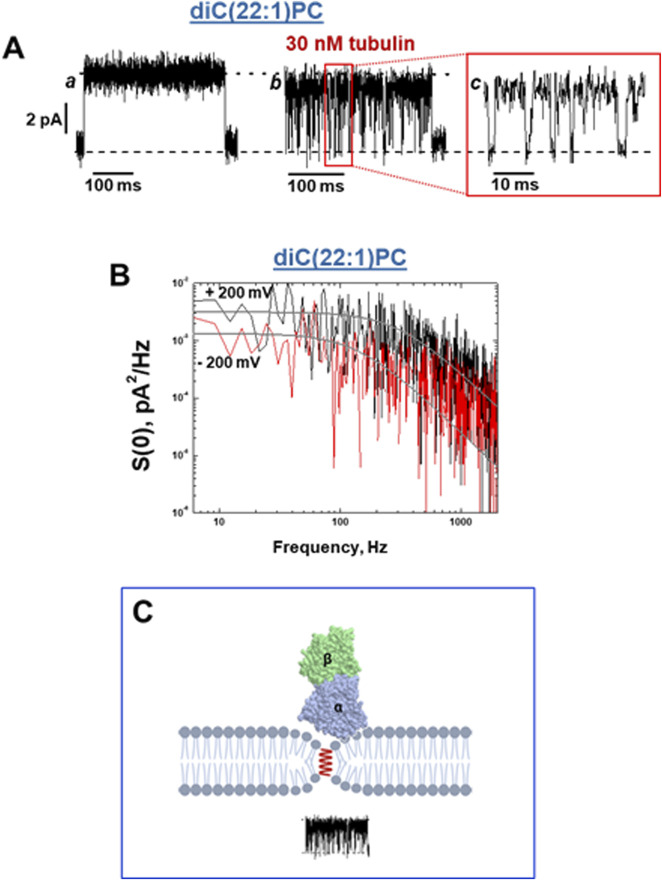
Tubulin induces fast blockage events down to zero-current in grA channels in a diC(22:1)PC membrane. **(A)** Current traces of a single grA channel in a diC(22:1)PC bilayer before (trace a) and after (trace b) addition of 30 nM tubulin to the cis compartment. The addition of tubulin induces rapid events of grA channel closure to a zero-current level, shown in trace c at a finer time scale. The applied voltage was 200 mV. The medium consisted of 1 M KCl buffered with 5 mM HEPES at pH 7.4. Current records were filtered with a digital eight-pole Bessel filter at 2 kHz. **(B)** The power spectral density of tubulin-induced current fluctuations depends on the polarity of the applied voltage. Solid lines represent the fits to Lorentzian spectra. **(C)** A cartoon of the local effect of tubulin on grA conductance. In the case of diC(22:1)PC membranes, binding of tubulin dimers is limited to the regions of membranes where headgroup packing is distorted by grA channel presence in the region of the lipid funnel forming the entrance to the channel. However, the integral properties of the membrane remain unchanged, and grA lifetime is unaltered. Adapted with permission from Rostovtseva et al. (2024). © 2024 by the authors. Licensee MDPI, Basel, Switzerland.

A characteristic effect of tubulin on grA channel is a generation of fast flickering channel conductance in DOPE (Figure 6A) and DPhPC membranes (Rostovtseva et al., 2024). Considering that the grA channel does not “gate” in the conventional sense used for ion channels gating, the origin of this puzzling phenomenon was investigated following a model proposed earlier by Armstrong and Cukierman (2002) and Ring (1986), where the intensity of grA channel flickering was related to the bilayer thickness. This hypothesis was tested recently (Rostovtseva et al., 2024) in experiments using the membranes formed from phosphatidylcholine with a C(22:1) unsaturated acyl chain (diC(22:1)PC), whose hydrophobic thickness is 0.76 nm larger than that of DOPC (Table 1). As shown in Figure 7A, in diC(22:1)PC membranes, the fluctuations between grA open and zero-conductance levels are well-pronounced and time-resolved only in the presence of tubulin. The corresponding power spectral densities (Figure 7B) show a noticeable asymmetry in current blockages with respect to the sign of the applied voltage. Such asymmetry is a result of one-sided tubulin addition (30 nM tubulin in the *cis* side) in these experiments. Similar asymmetry was also observed in DPhPC and PC/PE membranes (Rostovtseva et al., 2024). A natural interpretation of this asymmetry is that the positive potential applied to the side of tubulin addition (*cis* side) pushes a positively charged α-tubulin membrane binding domain (Figure 5) towards the membrane plane, leading to a higher power spectral density of tubulin-induced current fluctuations at positive voltage than at negative (+200 vs. −200 mV in Figure 7B). Notably, the highest spectral density, which corresponded to the highest frequency of blockage events, was obtained in the thickest diC(22:1)PC bilayers. Following the interpretation of grA conductance flickering phenomena proposed by Armstrong and Cukierman (2002), the current blockages could be understood as the result of tubulin-induced modulations of the lipid funnel that forms the channel entrance (cartoon in Figure 7C). Alternatively, the tubulin dimer diffusing along the membrane surface could transiently block the channel conductance while approaching the lipid funnel. Interestingly, when instead of full-length tubulin, a synthetic peptide comprising the helix H10 of α-tubulin–the identified membrane-binding tubulin domain (Figure 5, see Section 2) – was used in grA experiments, no effect on channel conductance or induction of current flickering was observed (Rostovtseva et al., 2024). At the same time, similar to the full-length tubulin, the synthetic peptide also increased grA lifetime, but less effectively (≈4 times increase) than tubulin, and in a much higher (μM) concentration range.

These results suggest that tubulin’s effect on channel conductance and the generation of rapid conductance blockages might have a different origin than the effect of tubulin on grA lifetime. In Section 3.2, we explored how tubulin-induced changes in *global* membrane properties affect grA lifetime: smaller PE headgroups, as compared with PC headgroups, appeared to be more prone to adjusting to the tubulin-induced redistribution of the packing forces towards lipid hydrocarbon chains. This flexibility of the PE headgroups provides conditions for stronger tubulin α-helix anchoring (Figures 2, 3, 5) causing the increase of grA lifetime (Figure 6). Notably, both full-length tubulin and the synthetic peptide reduce the bilayer deformation energy contribution of grA channel formation reflected in an increased lifetime.

In the case of diC(22:1)PC membranes, binding of tubulin dimers is limited to the regions of membranes where headgroup packing is distorted (less dense, analogous to a smaller headgroup) in the lipid funnel formed by the grA channel. This *local* change in the membrane properties leads to stable binding of tubulin only where grA channels have formed (pathways §4 in Figure 1). The limitation to the region of the lipid funnel leads to the virtually unchanged global properties of the membrane and thus unchanged grA lifetime; however, the localized binding is clearly manifested via transient channel blockages by the bulky body of the tubulin dimer. The absence of conductance flickering in the presence of the α-tubulin membrane binding peptide is not surprising considering its drastically smaller size of 14 kDa compared with a 100 kDa tubulin globule (Figure 7C) and, therefore, its inability to induce local modulations near the channel entrances. In relatively thin DOPC membranes, there is no measurable tubulin-induced flickering; by contrast, in thicker diC(22:1)PC membranes (Table 1), the flickering is most pronounced. These results show that the deeper grA is embedded into the bilayer, the larger the effect of tubulin on channel conductance and flickering. This makes the possibility of direct tubulin-grA interactions in our experiments extremely unlikely and points towards local grA-induced lipid packing defects as the sites of tubulin binding and the source of current fluctuations.

We can conclude that the tubulin-grA interaction studied here is an example of a complex phenomenon in which protein binding and protein-protein interactions are regulated by lipids. Both the binding of APs and the incorporation of integral proteins into the membrane alter its properties and, via this alteration, protein function. Moreover, it is natural to expect that the effect of tubulin binding on membrane mechanics is reciprocal.

## 5 Effect of α-synuclein on the lipid membrane properties, as reported by the grA lifetime

To further test our model of separation between global and local effects of AP on lipid membranes and embedded IMPs, we used another well-studied AP, αSyn. αSyn is a small, 14 kDa intrinsically disordered neuronal protein highly expressed in the central nervous system and constituting up to 1% of total cytosolic proteins in normal brain cells (Kruger et al., 2000). It is mostly known as a major component of the Lewy bodies found in the brains of Parkinson’s disease (PD) patients (Spillantini et al., 1997), the inclusions that are a pathological hallmark of this neurodegenerative disorder (Strohaker et al., 2019). Despite the obvious structural, functional, and genetic differences between αSyn and tubulin, they both belong to the AP family and are transiently associated with the cellular membrane. Similar to tubulin, αSyn was also found associated with mitochondrial membranes *in vivo* (Li et al., 2007; Nakamura et al., 2008; Nakamura, 2013; Robotta et al., 2014; Rovini et al., 2020) and with model membranes *in vitro* (Bodner et al., 2009; Pfefferkorn et al., 2012). Despite the obvious differences between tubulin—a compactly folded 110-kDa globular protein with a well-defined crystal structure (Nogales et al., 1998)—and αSyn—an unstructured 14-kD polypeptide—both proteins have one similarity in their structures: a disordered, highly negatively charged CTT. When tubulin or αSyn is added to the planar membrane bathing solutions with reconstituted VDAC, they both induce characteristic blockages of channel conductance (Rostovtseva et al., 2017; Rostovtseva et al., 2021). The CTTs of either tubulin or αSyn are the domains responsible for the reversible blockage of the VDAC pore. It was shown that when CTT of either protein is captured by VDAC pore, its selectivity is reversed to cationic, which is opposite to the anion-selective open state (Gurnev et al., 2011; Hoogerheide et al., 2017; Hoogerheide et al., 2018). Notably, the first step of both proteins’ interaction with VDAC is their transient binding to the membrane, followed by a partial and reversible block of the VDAC pore by their anionic CTTs driven by the applied potential. Therefore, the membrane binding of αSyn, as that of tubulin, has an array of physiological implications.

Due to the apparent importance of αSyn in neurodegeneration, an impressive amount of biophysical studies were devoted to αSyn interaction with membranes (Eliezer et al., 2001; Ulmer et al., 2005; Fusco et al., 2014; Fusco et al., 2017). One of the fascinating features of this small protein is that while being disordered in bulk solution, upon binding to the lipid membrane, αSyn’s N-terminal domain adopts a helical structure (Eliezer et al., 2001; Ulmer et al., 2005; Pfefferkorn et al., 2012; Fusco et al., 2014) with preferential binding to anionic lipids. αSyn also has a pronounced binding affinity to zwitterionic lipids (O'Leary et al., 2018; Jacobs et al., 2019) with a preference non-lamellar lipids, such as PE (Jo et al., 2000). Its membrane binding has a strong sensitivity to the membrane curvature and induces membranes remodeling, such as tubulation (Jiang et al., 2013). In summary, lipids with small and/or negatively charged headgroups enhance both binding affinity and helix formation of αSyn’s N-terminal membrane-binding domain. Therefore, αSyn seems to be an excellent AP candidate for testing its ability to modify lipid bilayer using a grA sensor probe.

**FIGURE 8:**
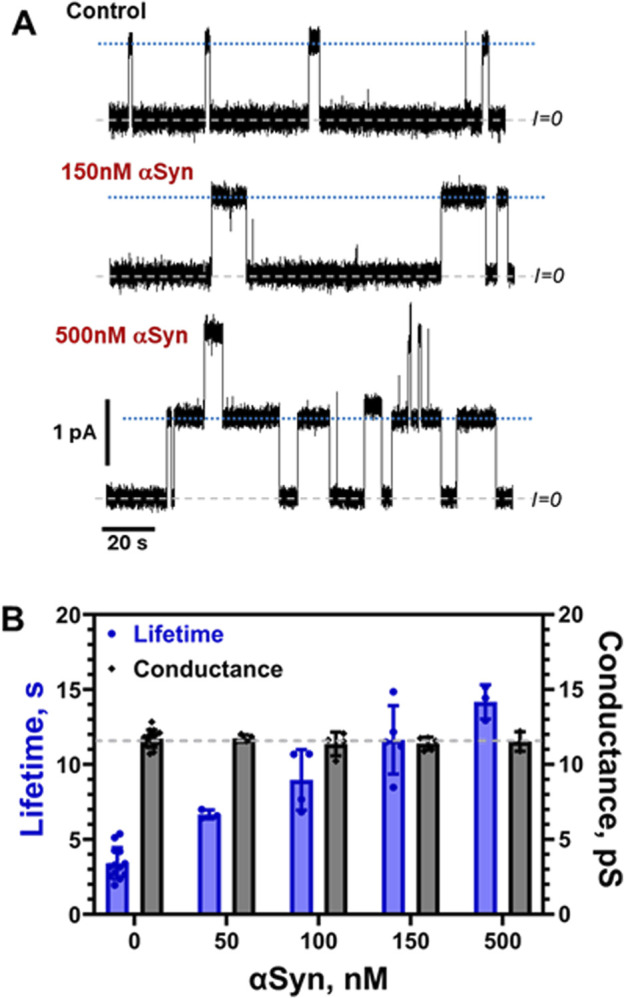
αSyn increases grA lifetime but does not change its conductance. **(A)** Current traces of the channels in a DPhPC membrane before and after the addition of 150 and 500 nM αSyn to the cis side of the membrane. Current records were filtered with a digital eight-pole Bessel filter at 50 Hz. Dashed lines indicate zero current level; dotted lines show a single channel current level. The membrane bathing solution contained 150 mM KCl buffered with 5 mM HEPES at pH 7.4. The applied voltage was 100 mV. **(B)** αSyn increases grA lifetime (blues bars) in a dose-dependent manner but does not change conductance appreciably (grey bars). The grey dashed line indicates average grA channel conductance at all conditions. Bars and error bars are the mean and standard deviation from the mean in 4–14 experiments.

The results of the effect of αSyn on grA parameters are shown in Figure 8. Similar to α-tubulin membrane-binding peptide (Rostovtseva et al., 2024), αSyn increases channel lifetime in a dose-dependent manner up to ≈5 times at 500 nM but does not measurably change grA conductance (Figure 8B). The concentration range at which αSyn affects grA is about 10 times higher than that of tubulin (Figure 6), but much lower than the micromolar range of α-tubulin synthetic peptide (Rostovtseva et al., 2024). We can conclude that αSyn affects the global properties of lipid membrane but, similarly to α-tubulin peptide, either does not sense the local membrane curvature near grA entrance, or, more likely, does not have a sufficiently bulky body to detectably disrupt ion flow through the channel. These findings naturally raise the question of whether large globular APs more effectively sense the local membrane curvature, which could be a subject of further studies.

## 6 Conclusion

Here we have established the reciprocal lipid-mediated interactions of an AP tubulin and an integral membrane protein grA in the absence of direct protein-protein interactions. The implications of these solely lipid-mediated interactions are broad, impinging on protein-membrane binding assays, the action of membrane proteins, and the mechanisms of action of membrane-altering small molecules.

First, these results suggest that membrane deformation can play a significant role in the binding of APs. In Section 2, we showed that tubulin binding to a lipid membrane, as observed by three different biophysical techniques, requires a multisite binding model to unify the individual observations. The presence of PE lipids, which alter the membrane properties to allow a greater degree of hydrophobic interactions, is important. Then, in Section 3, we showed that tubulin modifies the global properties of the membrane, which is expected in turn to alter tubulin’s binding propensity. These observations imply that, when describing the mechanics of binding, not only must the energetics of the multiplicity of binding sites be accounted for, but also the energy of membrane alteration. The latter is a collective, presumably protein sequence-dependent, effect.

Second, the lipid-mediated interactions between IMPs and their AP partners analyzed here provide a pathway by which the interactions between these proteins can be modulated *in vivo*. For example, the voltage-gating properties of VDAC are not significantly affected by the presence of tubulin with truncated CTTs (Rostovtseva et al., 2008). However, considering that the lipid composition of mitochondrial membranes is dynamic, especially under conditions of oxidative stress (Crimi and Esposti, 2011), or apoptosis (Kagan et al., 2004; Paradies et al., 2011), we can suggest that mitochondrial membrane remodeling modulates tubulin binding and, therefore, its regulatory interaction with VDAC. Because mitochondrial membranes are known to undergo dynamic remodeling in many cellular processes such as cell proliferation in disease and healthy development, we speculate that this type of regulation could be particularly relevant to other mitochondrial membrane proteins.

Finally, some membrane-altering small molecules, such as anesthetics, tricyclic antidepressants, and psychedelics, have properties similar to APs (Kapoor et al., 2019; Castellanos et al., 2020). The recognition of lipid-mediated protein-protein interactions could be instrumental in understanding the off-target action of some of these drugs on cell and organelle membranes and, therefore, on membrane proteins residing in, or peripherally associated with, those membranes.
